# Effects of ingesting a pre-workout supplement with and without synephrine on cognitive function, perceptions of readiness to perform, and exercise performance

**DOI:** 10.1186/1550-2783-11-S1-P36

**Published:** 2014-12-01

**Authors:** M Cho, YP Jung, C Goodenough, A O’Connor, R Dalton, K Levers, E Galvan, N Barringer, F Ayadi, J Carter, M Koozechian, S Simbo, A Reyes, B Sanchez, A Coletta, C Rasmussen, R Kreider

**Affiliations:** 1 Texas A&M University, College Station, Texas, USA

## Background

A number of nutritional strategies have been developed to optimize nutrient delivery prior to exercise. As a result, a number of pre-workout supplements have been developed to increase energy availability, promote vasodilation, and/or positively affect exercise capacity. The purpose of this study was to examine the acute effects of ingesting a pre-workout dietary supplement with and without synephrine on cognitive function, perceptions of readiness to perform, and exercise performance.

## Methods

In a double-blind, crossover, randomized and placebo-controlled manner; 25 apparently healthy and recreationally active men and women (21.76±3.00 yr, 15.24±5.26% fat, 25.09±3.03 kg/m2) volunteered to participate in this study and performed a Stroup-Color cognitive function test (CFT) and rated perceptions of readiness to perform on a visual analogue scale (RTP-VAS). Participants then ingested in a randomized and counterbalanced manner a dextrose flavored placebo (P); a pre-workout supplement (PWS) containing 3.0 g beta alanine, 2 g creatine nitrate, 2 g arginine AKG, 300 mg of N-acetyl tyrosine, 270 mg caffeine, 15 mg of Mucuna pruriens; or, the PWS with 20 mg of synephrine (PWS+S). Approximately 30 minutes following ingestion of the supplements, participants performed a second CFT, completed a RTP-VAS, and then performed 3 sets of 10 repetitions at 70% of 1 repetition maximum (1RM) on the bench press and leg press with 2 minutes recovery between sets and 5 minutes recovery from exercise modes. Participants completed as many repetitions as possible during the final set. Following a 5-minute recovery, subjects also performed a 30-sec Wingate Anaerobic Capacity test on a cycle ergometer for determination of peak power (PP), mean power (MP), and total work (TW). Lastly, subjects performed a third CFT and RPT-VAS test. Participants repeated the experiment after a one week washout period with alternate supplements provided in a randomized and counterbalanced manner. Data were analyzed by repeated measure MANOVA or ANOVA and are presented as means ± SEM from baseline. Consent to publish the results was obtained from all participants.

## Results

Repeated measures MANOVA analysis revealed significant interactions among supplementation groups in ratings of “I am optimistic about my future performance" (P: 3.70±0.95; PWS: 4.05±0.73; PWS+S: 4.21±0.63; p<0.01), “I feel vigorous and energetic” (P=3.35±0.91; PWS: 3.77±0.79; PWS+S: 3.89±0.74; p=0.01), and “I have little muscle soreness” (P=3.42±1.00; PWS: 3.81±1.36; PWS+S: 3.27±1.29, p=0.04) 30 minutes after ingestion. MANOVA revealed an overall Wilks’ Lambda time (p<0.001) and time x group interaction (p=0.003) effect on the CFT results. Delta analysis revealed that mean changes in word (P=0.64±1.1; PWS: 3.57±1.1; PWS+S: 7.40±1.1; p=0<0.001), color (P=1.41±0.7; PWS: 4.01±0.7; PWS+S: 5.08±0.7; p=0.002), and word-color (P=1.8±1.0; PWS: 3.28±1.0; PWS+S: 5.41±1.0; p=0.03) were greater in the PWS and PWS+S groups than P with PWS+S word responses greater than PWS. There were no significant differences among groups in Wingate PP (P: 1,579±510; PWS: 1,502±561; PWS+S: 1,491±516 W; p=0.46), MP (P: 602±132; PWS: 596±145; PWS+S: 583±188 W; p=0.60), or TW (P: 17,663±4,605; PWS: 17,850±4,341; PWS+S: 18,203±4,658 J; p=0.49). MANOA revealed significant Wilks’ Lambda time and time x group (p<0.001) effects in bench press and leg press lifting volume in the final set of exercise. MANOVA univariate analysis revealed no significant Greenhouse-Geisser differences among groups in third set bench press lifting volume (P: 4,749±1,606; PWS: 4,889±1,614; PWS+S: 4,870±2,000 lbs; p<0.51). Leg press lifting volume differed among groups (P: 27,607±9,608; PWS: 28,905±9,859; PWS+S: 19,342±4,855 lbs; p<0.51) but PWS supplementation did not provide greater benefit than P.

## Conclusion

Ingesting a PWS and PWS with 20 mg of synephrine 30-minutes prior to exercise enhanced perceptions of readiness to perform and cognitive function with no significant effects on anaerobic capacity or isotonic lifting volume.

